# Clinical balance assessment: perceptions of commonly-used standardized measures and current practices among physiotherapists in Ontario, Canada

**DOI:** 10.1186/1748-5908-8-33

**Published:** 2013-03-20

**Authors:** Kathryn M Sibley, Sharon E Straus, Elizabeth L Inness, Nancy M Salbach, Susan B Jaglal

**Affiliations:** 1 Toronto Rehabilitation Institute- University Health Network, Toronto, Canada; 2 Department of Physical Therapy, University of Toronto, Toronto, Canada; 3 Li-Ka-Shing Knowledge Institute, St. Michael’s Hospital, Toronto, Canada; 4 Faculty of Medicine, University of Toronto, Toronto, Canada; 5 Graduate Department of Rehabilitation Sciences, University of Toronto, Toronto, Canada

## Abstract

**Background:**

Balance impairment is common in multiple clinical populations, and comprehensive assessment is important for identifying impairments, planning individualized treatment programs, and evaluating change over time. However, little information is available regarding whether clinicians who treat balance are satisfied with existing assessment tools. In 2010 we conducted a cross-sectional survey of balance assessment practices among physiotherapists in Ontario, Canada, and reported on the use of standardized balance measures (Sibley et al. 2011 Physical Therapy; 91: 1583-91). The purpose of this study was to analyse additional survey data and i) evaluate satisfaction with current balance assessment practices and standardized measures among physiotherapists who treat adult or geriatric populations with balance impairment, and ii) identify factors associated with satisfaction.

**Methods:**

The questionnaire was distributed to 1000 practicing physiotherapists. This analysis focuses on questions in which respondents were asked to rate their general perceptions about balance assessment, the perceived utility of individual standardized balance measures, whether they wanted to improve balance assessment practices, and why. Data were summarized with descriptive statistics and utility of individual measures was compared across clinical practice areas (orthopaedic, neurological, geriatric or general rehabilitation).

**Results:**

The questionnaire was completed by 369 respondents, of which 43.4% of respondents agreed that existing standardized measures of balance meet their needs. In ratings of individual measures, the Single Leg Stance test and Berg Balance Scale were perceived as useful for clinical decision-making and evaluating change over time by over 70% of respondents, and the Timed Up-and-Go test was perceived as useful for decision-making by 56.9% of respondents and useful for evaluating change over time by 62.9% of respondents, but there were significant differences across practice groups. Seventy-nine percent of respondents wanted to improve their assessments, identifying individual, environmental and measure-specific barriers. The most common barriers were lack of time and knowledge.

**Conclusions:**

This study offers new information on issues affecting the evaluation of balance in clinical settings from a broad sample of physiotherapists. Continued work to address barriers by specific practice area will be critical for the success of any intervention attempting to implement optimal balance assessment practices in the clinical setting.

## Background

Balance, defined as the ability to keep the center of mass within the base of support [1], is a critical skill required for many functional activities, such as mobility and fall avoidance [2,3]. Balance impairment is common across a range of populations, including, but not limited to: stroke [4], brain injury [5], arthritis [6], and up to 75% of people aged 70 years and older [7]. Fortunately, in many of these populations balance can be improved through treatment that emphasizes exercise [2,8,9], and under optimal conditions (i.e. moderate- high balance challenge [10]) can reduce risk and rate of falls [11].

In light of the potential to improve balance with treatment, comprehensive assessment of postural control is recommended in order to identify specific impairments and develop individualized treatment plans [12], and the use of valid and reliable tests to do so is a recognized component of evidence based practice [13]. Treatment of balance impairments is an important focus of physiotherapy practice [14], and previous work has demonstrated that physiotherapists (PTs) frequently use standardized measures in their assessments [15]. However, it has also been reported that PTs do not always “trust” the results of such measures [16], suggesting that available standardized measures may not meet the needs of individuals working in clinical practice.

The long-term objective of this work is to optimize balance assessment in clinical practice, in order to identify relevant impairments, inform treatment planning, and in turn, maximize the effectiveness of exercise interventions to improve balance and reduce falls in high risk populations. We have previously reported initial findings from a 2010 survey of PTs practicing in Ontario, Canada [17], in which most respondents regularly used at least one standardized measure in their assessment of balance. The most commonly used measures were the Single Leg Stance Test [18], Timed Up-and-Go (TUG) test [19] and Berg Balance Scale [20]. However, the most commonly used method of balance assessment overall was non-standardized movement observation. We also found that most respondents regularly evaluated many components of balance, but some important components related to fall risk were not regularly assessed by most PTs. In particular, reactive postural control – postural reactions following a loss of balance induced by external perturbations [21,22] – was regularly assessed by less than half of respondents. These results suggest that there may be gaps in comprehensive assessment of balance and use of standardized measures in clinical decision-making.

However, prior to developing an intervention to address such gaps, it is important to understand the perceptions and needs of the end-users (in this case, the practicing clinicians) as they may identify barriers and facilitators to engaging in optimal practices [23]. The objective of this analysis was to examine additional survey data that explored respondents’ perceptions of their balance assessment practices and determine 1) general perceptions about balance assessment in physiotherapy practice; 2) perceived utility of existing standardized balance measures and compare perceptions across areas of clinical practice; 3) satisfaction with the current ability to evaluate standing balance and reactive postural control; and 4) factors influencing satisfaction with standing balance and reactive postural control assessment.

## Methods

### Overview of the survey

This study received ethics approval from the University of Toronto. Details of the survey design and questionnaire have been previously reported [17]. In brief, 1000 practicing PTs who specified orthopaedics, neurology, rehabilitation, health promotion/prevention or general as the primary area of practice in adult or geriatric populations were randomly sampled from the registry of provincial regulatory body, the Ontario College of PTs. A modified Dillman 3-step mailing approach was used [24], in which the survey package was mailed in March 2010, followed by a reminder postcard 4 weeks later, and a complete survey package to non-responders 4 weeks after that. PTs were asked to complete the questionnaire only if they treated people with balance impairments. PTs who did not treat people with balance impairments were ineligible and asked to return the questionnaire (indicated by answering no to the first question and returning the rest of the questionnaire blank), as well as those who declined to participate (indicated by returning a blank questionnaire).

Prior to distribution, the survey was pilot tested for sensibility (face and content validity and comprehensibility) with a convenience sample of 12 PTs who represented varied patient populations and practice settings. In addition to collecting information about demographics, practice setting, frequency of assessment of eight components of balance and use of eight standardized balance measures [18-20,25-29] (published previously), the questionnaire probed perceptions about several aspects of balance assessment. General perceptions of standardized measurement and existing balance measures were explored in four statements that addressed the importance of measurement, the adequacy and comprehensiveness of existing balance measures, and the ability of current measures to meet respondents’ clinical needs. Respondents rated agreement with these statements on a 5-point Likert scale with descriptive anchors (strongly agree, agree, neutral, disagree, strongly disagree). Perceived utility of eight existing standardized balance measures (Balance Evaluation Systems Test [BESTest], Berg Balance Scale, Clinical Test of Sensory Integration in Balance [CTSIB], Community Balance and Mobility [CB&M] Scale, Performance Oriented Mobility Assessment [POMA], Push & Release test, Single Leg Stance test, TUG test was determined by asking respondents to rate the utility of each measure for 1) informing clinical decision-making and choice of treatment, and 2) evaluating change in balance over time. Utility was rated on a 5-point Likert scale with descriptive anchors (extremely useful, somewhat useful, neutral, somewhat not useful, not at all useful) and there was an option to indicate if the respondent was not familiar with the measure. Satisfaction with current balance assessment practices was probed through two questions asking respondents whether they wanted to improve their assessment of 1) standing balance (defined as on-going center of mass control when the base of support does not change) and 2) reactive postural control (defined as responses to external perturbations). In each case, if a respondent indicated that they wanted to improve their ability to assess balance, they were asked to identify factors influencing their ability to assess balance in the past, by selecting any of nine relevant personal, organizational or systemic barriers noted throughout the literature from a closed-ended list. There was also an open-ended option for additional factors. If a respondent indicated they did not want to improve their approach to balance assessment, they were asked to indicate why not from selecting from a unique closed-ended list of six personal, organizational or systemic barriers, with an open-ended option.

### Data analysis

The Likert ratings for general perceptions of existing measures were collapsed into three categories: “agree” (combining the “strongly agree” with the “agree” category), “neutral”, and “disagree” (combining the “disagree” with the “strongly disagree” category). For each of the measures surveyed, the Likert scale ratings for utility also were collapsed into four categories: “useful” (combining “extremely useful” and “somewhat useful”), “neutral”, “not useful” (combining “somewhat not useful” and “not at all useful”), and “not familiar”.

Descriptive statistics (frequency and percentage) were used to summarize all variables. Preliminary analysis revealed that for five of the eight measures (BESTest, CTSIB, CB&M, POMA, Push and Release), the majority of respondents (25-74%) indicated they were unfamiliar with each measure and did not rate clinical utility. Given the absence of utility data for those measures, subsequent analysis focused on the utility of Single Leg Stance, TUG and Berg tests (the top three measures used by respondents [17]). The proportion of respondents rating each measure as “useful” for clinical decision making and evaluating change over time was compared between respondents working across four clinical practice areas (orthopaedics, neurology, geriatrics, and “general” rehabilitation [comprised of multiple and/or complex conditions from a combination of the other practice areas]) with Chi-squared tests, using predicted proportions for the expected values. Data analysis was conducted using Statistical Analysis Software (SAS), version 9.1.

## Results

As reported previously, of the 1000 questionnaires distributed, 509 were returned and 369 individuals (72.5%) completed the questionnaire. Demographic and practice setting characteristics have also been reported [17]; in brief: most respondents were female (77.2%), between 31 and 40 years old (32.5%), and held a Bachelor’s entry-to-practice degree (70.2%). The greatest proportion of respondents worked in orthopaedics (46.3%), while 21.4% worked in neurology, 7.9% worked in geriatrics, and 24.4% worked in general rehabilitation. Forty eight percent of respondents worked with both adult and geriatric (>65 years), while the remainder worked with either adult (23.3%) or geriatric populations only (28.7%). The greatest proportion of respondents worked with that population for more than 10 years (45.5%), in private outpatient clinics (32.5%), in urban (59.1%), and inter-professional (70.5%) settings.

General perceptions of standardized measurement and existing balance measures are reported in Table 1. Overall, 347 respondents (94.0%) agreed that quantifying impairments and outcomes with measurement is important for patient care. However, only 160 respondents (43.4%) agreed that existing standardized balance measures meet their practice needs, 109 (29.5%) agreed that existing measures adequately quantify balance impairment at all levels of severity, and 78 (21.1%) agreed that existing measures comprehensively evaluate all aspects of balance.

**Table 1:** General perceptions of standardized measurement and existing balance measures

	**Agree**		**Neutral**		**Disagree**		**n missing**
**Statement**	**n**	**%**	**n**	**%**	**n**	**%**	
Quantifying impairments and outcomes with measurement is important for patient care.	347	94.0	14	3.8	6	1.6	2
Existing standardized balance measures meet my practice needs.	160	43.4	94	25.5	106	28.7	9
Existing measures adequately quantify balance impairment at all levels of severity.	109	29.5	133	36.0	120	32.6	7
Existing measures comprehensively evaluate all aspects of balance.	78	21.1	144	39.0	140	37.9	7

Perceived utility of the three most commonly used standardized balance measures are reported in Table 2. Over 70% of respondents indicated that the Single Leg Stance and Berg tests were useful for clinical decision making and evaluating change over time, while the TUG test was perceived as useful for decision making by 56.9% of respondents and useful for evaluating change over time by 62.9% of respondents. The proportion of respondents rating each measure as “useful” in each area of clinical practice is reported in Table 3. There were significant between-group differences in the proportion of useful ratings for evaluating change over time for all three commonly used measures, and significant between-group differences in the proportion of useful ratings for decision making for the Single Leg Stance and Berg tests (all p < = 0.005).

**Table 2:** Perceived utility of commonly-used balance measures

	**Useful**		**Neutral**		**Not useful**		**Not familiar with measure**		**n missing**
	**n**	**%**	**n**	**%**	**n**	**%**	**n**	**%**	
**Utility for clinical decision-making**									
Single leg stance	305	82.7	26	7.0	17	4.6	2	0.5	19
Berg	269	72.9	28	7.6	34	9.2	23	6.2	15
TUG	210	56.9	53	14.4	56	15.2	27	7.3	23
**Utility for evaluating change over time**									
Single leg stance	281	76.2	31	8.4	15	4.1	3	0.8	39
Berg	265	71.8	27	7.3	20	5.4	22	6.0	35
TUG	232	62.9	35	9.5	31	8.4	24	6.5	47

**Table 3:** Proportion of respondents who rate measures as “useful”, by practice area

	**Orthopaedics (n = 171)**			**Neurological (n = 79)**			**Geriatric (n = 29)**			**General rehab (n = 90)**				
	**n**	**%**	**n missing**	**n**	**%**	**n missing**	**n**	**%**	**n missing**	**n**	**%**	**n missing**	***X***^**2**^	**p**
**Useful for clinical decision-making**														
Single leg stance	156	91.2	8	62	78.5	3	18	62.1	5	70	77.8	3	21.7	<0.0001
Berg	100	58.5	13	75	94.9	0	21	72.4	0	74	82.2	2	16.9	0.0007
TUG	79	46.2	12	52	65.8	6	20	69.0	2	59	65.6	3	4.5	0.2
**Useful for evaluating change over time**														
Single leg stance	145	84.8	14	57	72.2	9	14	48.3	8	65	72.2	8	15.6	0.0014
Berg	99	57.9	18	69	87.3	5	21	72.4	4	76	84.4	8	20.7	<0.0001
TUG	85	49.7	22	60	75.9	11	20	69.0	5	67	74.4	9	12.8	0.005

Two hundred ninety-three respondents (79.4%) reported wanting to improve assessment of standing balance, while 308 (83.5%) wanted to improve assessment of reactive postural control. Among respondents who reported wanting to improve assessment, the factors influencing their ability to do so are reported in Tables 4 and 5. The top three barriers affecting respondents who wanted to improve standing balance assessment were lack of time (61.8%), lack of knowledge (44.4%) and inappropriate tools for the respondents’ patient population (39.2%). The top three barriers affecting respondents who wanted to improve assessment of reactive postural control were lack of knowledge (57.7%), lack of time (46.4%), and lack of availability of tools (38.6%). The factors influencing respondents who did not want to improve their ability to assess balance are reported in Table 6. The top 3 barriers influencing both standing balance and reactive postural control were the perception that valid tools already exist, low priority and lack of time.

**Table 4:** Factors influencing respondents who want to better assess standing balance (n = 293)

**“What has influenced your ability to assess standing balance in the past?”**	**n**	**%**
Lack of time	181	61.8
Lack of knowledge	130	44.4
Tools not appropriate for population	115	39.2
Tools not available	84	28.7
Sensitivity to change	81	27.6
Lack of equipment	76	25.9
Lack of consensus on what to assess	60	20.5
Low priority	41	14.0
Tools difficult to administer	35	11.9
Patient environment	4	1.4
Unaware of tools available	2	0.7
Client not interested in test	1	0.3
Patient cognition	1	0.3
Patient pain	1	0.3
Lack of personnel	1	0.3
Confidence in administering tests	1	0.3
Already doing a good job	1	0.3
Clinical relevance	1	0.3

**Table 5:** Factors influencing respondents who want to better assess reactive postural control (n = 308)

**“What has influenced your ability to assess reactive postural control in the past?”**	**n**	**%**
Lack of knowledge	178	57.8
Lack of time	143	46.4
Tools not available	119	38.6
Tools not appropriate for population	80	26.0
Lack of equipment	76	24.7
Lack of consensus on what to assess	52	16.9
Sensitivity to change	46	14.9
Tools difficult to administer	35	11.4
Low priority	10	3.2
Patient environment	4	1.3
Beliefs about assessment	3	1.0
Patient cognition	1	0.3
Patient pain	1	0.3
Lack of personnel	1	0.3
Confidence in administering tests	1	0.3
Confidence about reliability of tests	1	0.3

**Table 6:** Factors influencing respondents who did not want to improve balance assessment

	**Standing balance (n = 72)**		**Reactive postural control (n = 55)**	
**“Why not?”**	**n**	**%**	**n**	**%**
Valid tools already	53	73.6	19	34.5
Low priority	10	13.9	19	34.5
Lack of time	9	12.5	14	25.5
Lack of knowledge	3	4.2	5	9.1
Lack of personal interest	2	2.8	2	3.6
Lack of support among colleagues	0		3	5.5

## Discussion

The results of this study offer new information on issues affecting the evaluation of balance in clinical settings from a broad sample of PTs. The findings have both specific implications for on-going efforts to optimize balance assessment tools and practices and wider implications for the field of implementation science. Respondents conveyed a mixed message about their satisfaction with current balance assessment practices: while the majority reported that the commonly used standardized balance measures were useful to them, they also indicated that existing measures did not meet their needs and wanted to improve multiple aspects of balance assessment.

The general dissatisfaction with current standardized balance measures is supported by previous work reporting that PTs prefer clinical instinct over standardized measures in clinical practice [16]. For example, one investigation of clinical decision-making in balance assessment by PTs noted that participants reported using standardized balance measures to quantify balance with an objective reference, but their clinical decisions were based on observations, not the findings of standardized measures [14]. And yet, we also note a paradox in our findings: most respondents also perceived specific measures – the Single Leg Stance, Berg and TUG tests – as useful for informing clinical decision-making and evaluating change over time (although there were significant variations in perceived utility across practice areas).

The reasons underlying the perceived clinical utility of these measures is not yet clear, as the survey was limited to exploring *what* respondents perceptions were, not *why* they held them. Additional study is needed to explore the essential components of clinical utility, by probing both general and specific features of individual measures and exploring how and why clinicians value them. There appears to be variability in how the concept of clinical utility is perceived. For example, in the current study the majority of respondents viewed all three commonly used measures as useful for evaluating change over time; in contrast, an expert PT panel that used a Delphi process to recommend purposes for specific standardized balance measures recommended only the Berg scale for evaluating change over time, and recommended the Single Leg Stance and TUG for identifying functional limitations and screening only [30]. However, it is also important to weigh individual clinician perceptions and usage against empirical data, as it has also been shown that each of these tests may have differences in sensitivity to change and/or floor or ceiling effects across populations [31-33]. Indeed, some of the differences in perceived utility across respondents working with different populations were appropriate given the published data. For example, the Single Leg Stance test received the fewest “useful” ratings for evaluating change over time among respondents working with neurological populations, consistent with data demonstrating that it is not sensitive enough to follow changes over time or after intervention in chronic stroke [32]. Given that the perceived clinical relevance of a measure is a known facilitator for adoption of best practices [34], improved understanding of the concept of clinical utility in measurement could be applied to other domains where there is also a recognized need for changes in practice, such as functional outcome measurement [35]. Furthermore, additional investigation exploring why clinicians do or do not find specific tests to be clinically useful is critical for both identifying where more psychometric studies are required and to inform how potential interventions should be developed.

Our examination of PTs’ desire to improved balance assessment practices is new to the literature. While the finding that the majority of respondents wanted to improve their assessment of standing and reactive postural control requires additional study to verify and explore in greater depth, the breadth of our study population and large sample size give credence to these results. Given our previous finding that reactive control was regularly assessed the least, the reported desire to improve reactive assessment practices was not surprising. However, the finding that so many respondents also wanted to improve standing balance was unexpected, given the satisfaction with the commonly-used measures and availability of resources to do so. Such findings illustrate the value of these research questions in informing the development of future interventions, as even when practices are good, end-users may still recognize the potential for improvement.

The desire to change assessment practices is an important motivational factor for behavior change, and one that has been regarded as a necessary prerequisite for action [36]. However, such motivation is not sufficient, as it is well recognized that there are a number of domains involved (at both individual and organizational levels) in changing behavior to implement evidence-based practice [37]. Indeed, despite their positive sentiment towards improving balance assessment practices, respondents identified a number of factors influencing their ability to change their practice, including individual (e.g. lack of knowledge, low priority), environmental (e.g. lack of time and personnel), and measure-specific barriers (e.g. tools not available, tools not appropriate for population). The most commonly cited factors were time and knowledge. Although both are commonly-reported barriers to evidence-based practice [38,39], the finding that lack of knowledge was the main issue affecting assessment of reactive postural control may partially explain our previously-reported finding that it was the least commonly assessed component of balance among our respondents. Some respondents expressed issues related to motivation (such as low priority and confidence with performing the assessments) but these were not factors for the majority of PTs. Respondents also identified issues with existing standardized balance measures and how this influenced their assessment abilities. For example, a number of respondents believed that the existing tools were not appropriate for their population or were not sensitive to change– which, as noted, has also been empirically determined for some standardized balance measures [32,33]. In contrast, some of the factors reported by respondents represent perceptions that are actually incorrect; for example, the third most-commonly reported barrier to assessing reactive postural control was the belief that tools are not available, when in fact there are several validated standardized measures for assessing it [25,40]. Indeed, the fact that most respondents reported they were unfamiliar with one of these measures (the BESTest) may have contributed to this perception. The issue of incorrect knowledge versus lack of knowledge highlights an important distinction for the development of knowledge translation interventions, both specific to balance assessment and across clinical practice.

Although the proportion of respondents who did not want to improve their assessment of balance was a minority, the opinions of these individuals are informative. The primary factor influencing assessment of standing balance in this group was the perception that valid tools already exist. This is arguably an appropriate justification as there are a number of standing balance measures validated for various purposes [30]. However, there were no clear majority reasons for not wanting to improve assessment of reactive postural control. It was perceived among over one third of respondents that valid measures already exist (which is true), but the survey findings also showed that most respondents were not using these measures [17]. Motivational factors in light of environmental constraints (lack of time and low priority) were perhaps the most salient issues.

The identification of barriers to using knowledge – in this case knowledge of optimal balance assessment – is a recognized component of knowledge translation theory [41] and important for adapting knowledge to local contexts and tailoring specific interventions to implement evidence-based practice. The identification of individual, environmental and measure-specific barriers influencing PTs’ ability to assess balance in this study represents an important first step towards the development of strategies to optimize assessment in clinical practice which may comprise potential interventions or warrant additional developmental research.

With respect to individual barriers, the lack of knowledge respondents expressed about how to evaluate reactive postural control might be addressed by educational interventions that focus on practical training about the standard measures available for evaluating reactive balance and how to administer them. For individuals who did not indicate a desire to improve reactive control assessment, an additional education component may be required which would describe the theory and rationale for considering this component of balance. However, it is also recognized that education alone is not sufficient to change practice [42], and more active strategies, such as a clinical champion in individual sites, or strategies informed by theories of behavior change [43], may have potential benefit. Current evidence for effective knowledge translation interventions in rehabilitation is limited [44,45], and future rigorously-designed studies evaluating the effectiveness of a targeted intervention to increase reactive postural control assessment will contribute to the fields of both fall prevention and implementation science. With respect to measure-specific barriers, respondents’ reports that balance assessment tools are not appropriate for the populations they work with highlights the need for additional development research to verify the psychometric properties of existing measures. Given the plethora of measures available and the recognition that consensus is needed on the use of standardized balance measures [35], efforts should focus on validating existing tools and developing optimized assessment protocols across clinical populations. Finally, with respect to environmental barriers and the need to address the perceived lack of time, we note the irony that this is one of the most-commonly reported barriers to behaviour change, but is one of the least targeted outcomes for intervention. Such efforts require both individual and organizational commitment, and targeting non-traditional health care stakeholders (such as middle managers [46]) may help address these issues.

There were some limitations in the study design that restrict the scope of the conclusions. As noted already, further inquiry by qualitative study will offer more in-depth explanations of the factors shaping respondents’ perceptions about balance assessment and the interactions between related factors. Due to space constraints in the questionnaire, we did not inquire about the desire to improve dynamic balance assessment during tasks such as gait, and we acknowledge the limitation of being unable to comment on this component of balance. Furthermore, our closed-ended list provided of potential factors influencing the desire to improve balance assessment practices was not exhaustive. Although the open-ended option that was included did not include any reasons cited by more than a few individuals, we cannot comment on other domains postulated to affect health professionals’ behavior (such as memory and attention [37]). The role of habit in clinical practice is receiving increased attention [47], and this is something we did not address in the survey. Our inability to reliably report on the clinical utility of five of the eight measures surveyed due to low familiarity with the measures is unfortunate – albeit telling. Finally, the results are specific to a relatively small geographic region (Ontario, Canada) and profession (PTs), so caution should be exercised when generalizing the results. In particular, there is a need to examine practices in community fall prevention programs, which may employ other professions (such as kinesiologists) and experience different barriers than PTs.

## Conclusions

This study makes an important contribution to balance, mobility and fall prevention research and implementation efforts by illustrating the recognition among physiotherapists in Ontario, Canada that there are gaps in an important area of their practice: assessment of balance abilities. This work establishes that the end-users (frontline physiotherapy clinicians) are aware of the practice gap and acknowledge that there is a need to address it. This is an important preliminary step towards optimizing clinical practice in balance assessment, and while certainly not sufficient, suggests that these end-users may be receptive to innovative assessment solutions, provided they meet user needs. This will be no small feat, as there are numerous barriers to overcome. Use of integrated knowledge translation strategies that continue to engage all relevant stakeholders, in particular, the end-users, will be important for feasible and sustainable implementation of optimal balance assessment practices to achieve the ultimate goal of improving balance to optimize mobility and reduce more falls.

## Abbreviations

PT: Physiotherapist; TUG: Timed up-and-go; BESTest: Balance evaluation systems test; CTSIB: Clinical test of sensory integration in balance; CB&M: Community balance and mobility scale; POMA: Performance oriented mobility assessment

## Competing interests

The authors declare that they have no competing interests.

## Authors’ contributions

All authors participated in the study design, interpretation of the results, and read and approved the final manuscript. KMS conceived of the study, coordinated data collection, analyzed the results and wrote the manuscript.
